# Editorial: *EHP* Evolution Continues

**Published:** 2007-06

**Authors:** Kenneth S. Korach, Steven R. Kleeberger, Matthew P. Longnecker, Dorothy L. Ritter

**Affiliations:** Interim Editor-in-Chief, *EHP*, E-mail: EHPEditor@niehs.nih.gov; Interim Deputy Editor, *EHP*; Interim Deputy Editor, *EHP*; Managing Editor, Mini-Monographs, *EHP*

In the May 2007 issue of *EHP*, we announced that we would cease publication of the Mini-Monograph series this year, instead publishing more Review articles on topics that might once have been covered by Mini-Monographs. Here, we explain that decision and give some historical perspective on the Mini-Monographs.

*EHP* was begun in 1972 by David P. Rall, then director of the NIEHS. In addition to publishing original research articles, the journal was intended as a means of publishing proceedings of conferences and workshops on environmental health sciences, a field then in its infancy. In 1992, with new NIEHS director Kenneth Olden at the helm, we began publishing the *EHP* you know today, and proceedings were published separately as *EHP* Supplements. In 2003, we moved from the Supplements format to the Mini-Monographs to better serve the changing needs of the environmental health science community with a more concise yet comprehensive publication. Each of these formats served the scientific community well at its respective time.

However, scientific publishing has been revolutionized in the past decade. With the explosive growth of the Internet, the introduction of online publishing, and the advent of electronic printing technology, it is critical that research findings be published as soon as possible. We believe that presenting environmental health sciences research in a Review format, rather than as Mini-Monographs, will meet the challenge of constantly changing publishing technologies and the changing needs of our readers; this Review format will also parallel a similar format used by a number of other leading scientific publications.

We also believe that this shift toward the Review format will enhance the integrity and quality of the journal. The very nature of this format allows for more current data to be presented concisely and as expeditiously as possible, resulting in a Review that is cited more often. This, in turn, increases the citability of the journal as a whole, which benefits both *EHP* and all authors submitting papers to the journal. Indeed, *EHP*’s status as the number-one environmental health science journal directly reflects how often its papers are cited.

We . . . believe that this shift toward the Review format will enhance the integrity and quality of the journal. The very nature of this format allows for more current data to be presented concisely and as expeditiously as possible.

Two more Mini-Monographs will be published in 2007, whereupon the series will end. We look forward to this continuing evolution of *EHP* into a stronger journal than ever.

## Figures and Tables

**Figure:**
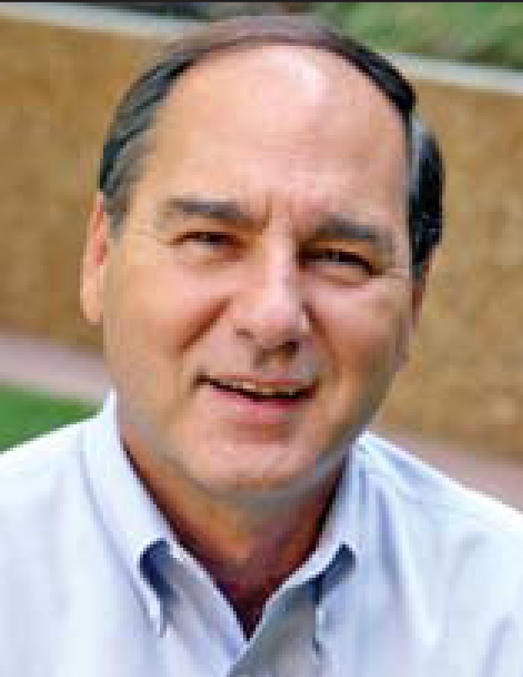
Kenneth S. Korach

**Figure:**
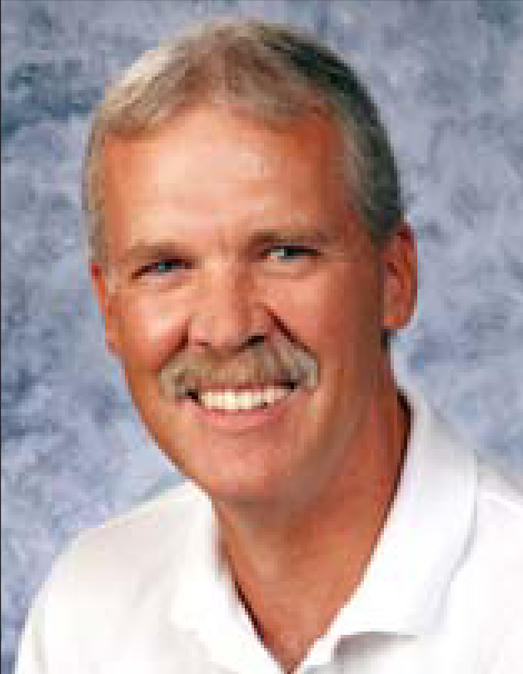
Steven R. Kleeberger

**Figure:**
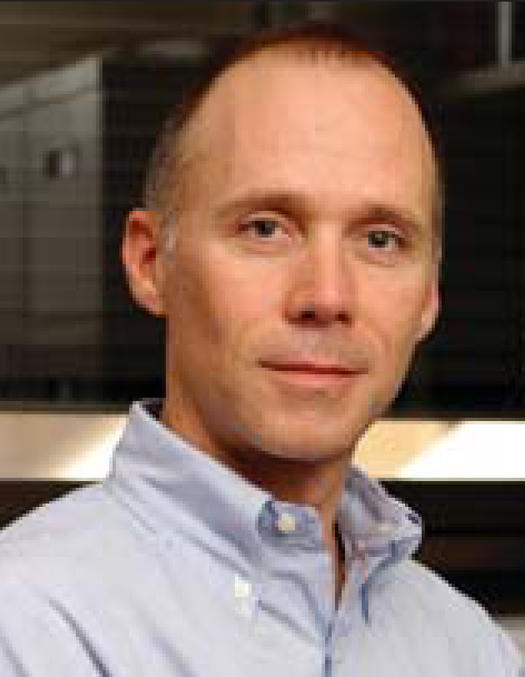
Matthew P. Longnecker

**Figure:**
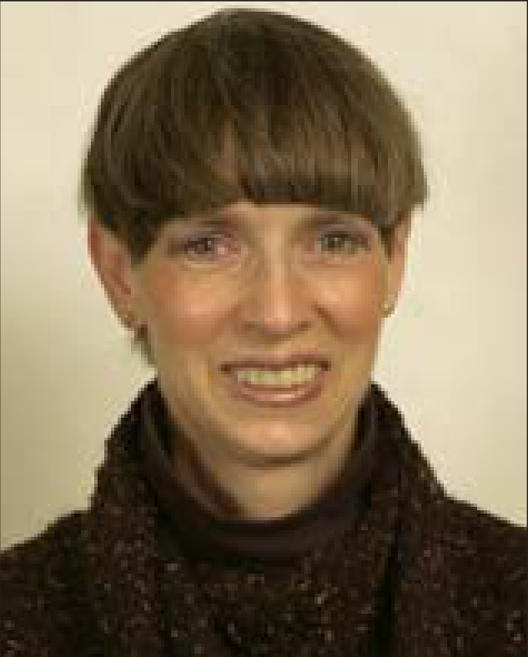
Dorothy L. Ritter

